# Extracellular Vesicle Release Within Energetic and Quantitative Constraints: Implications for Function

**DOI:** 10.1002/jev2.70340

**Published:** 2026-07-13

**Authors:** Christian Preußer, Elke Pogge von Strandmann

**Affiliations:** ^1^ Marburg University EV‐iTEC Core Facility Marburg Germany; ^2^ Marburg University Institute for Tumor Immunology Marburg Germany

**Keywords:** cargo loading, extracellular vesicles, proteostasis, thermodynamics

## Abstract

Extracellular vesicles (EVs) are widely framed as dedicated mediators of intercellular communication. Several studies attribute signalling and regulatory functions to vesicle‐associated cargo. However, functional interpretation is frequently inferred from detection and correlation, whereas quantitative and energetic boundary conditions remain underexamined. Here, an alternative but non‐exclusive perspective is proposed: EV release is considered as a regulated consequence of membrane turnover and proteostatic balance in cells operating far from thermodynamic equilibrium. Vesicle formation represents an energetically permissible transition within a constrained membrane system. Communication may arise from this process, but it should not be presumed as its primary rationale. Any EV‐mediated effect requires sequential steps such as encounter, uptake, cytosolic access, and sufficient cargo copy number, with each imposing limiting thresholds. At low mean copy numbers, stochastic partitioning and systemic dilution constrain reproducible impact. Enrichment alone does not establish functional sufficiency. This system does not deny EV function. It situates functional claims within explicit quantitative, statistical, and energetic boundary conditions and formulates experimentally testable criteria for their validation.

## Graphical abstract

Extracellular vesicle release reflects a thermodynamic membrane baseline in cells maintained far from equilibrium. Cargo loading follows stochastic partitioning, so most vesicles contribute to flux while only a minority can exert function; biological impact arises only when sequential quantitative thresholds collectively exceed stochastic and dilution constraints.

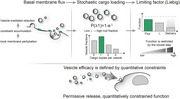

## 1 Introduction

Since their initial description by Chargaff and West (1946) and the subsequent, widely regarded characterization by Wolf (1967) as “platelet dust,” extracellular vesicles (EVs) have been recognized as integral components of biological systems. Nearly all examined cell types constitutively release membrane‐bound particles, a process strongly influenced by cellular state and stress. Following the discovery by Raposo et al. (1996), Ratajczak et al. (2006), and Valadi et al. (2007) that EVs can act as effective transport vehicles, research in this field expanded considerably in the late 2000s as the role of EVs in cell‐cell communication became more established. The prevalence and functional diversity of EVs remain subjects of debate, as numerous functions have been attributed to them, not all of which can be simultaneously realized. The frequent detection of EV‐associated molecules and correlated phenotypes is commonly interpreted as evidence of functional significance. For example, the co‐occurrence of tumour‐derived EVs carrying oncogenic proteins and increased metastasis in recipient tissues is often interpreted as functional evidence, although this alone remains correlative.

However, this approach mixes correlation with causation and may overlook essential factors such as stoichiometry, concentration thresholds, and kinetic constraints. To properly address this issue, it is essential to establish clear criteria for causal proof of EV functions. This would involve demonstrating specific mechanistic pathways, meeting quantitative thresholds, and accounting for the broader energetic and organizational context in which these biophysically induced phenomena occur, thereby providing a basis for functional claims (Welsh et al. 2024). Community efforts toward methodological standardization and reporting transparency, including the MISEV guidelines (Lötvall et al. 2014; Théry et al. 2018; Welsh et al. 2024), have improved technical rigor and inter‐study comparability. Yet standardization cannot resolve whether measured cargo quantities exceed the stoichiometric and kinetic thresholds required for functional relevance under physiological conditions.

Here, an alternative perspective is discussed that the release of EVs primarily regulates membrane turnover and proteostasis within the cell, which operates as an open, energy‐dissipating system. Although this view does not exclude the possibility of functional intercellular communication, it questions the assumption that communication is the primary evolutionary or mechanistic rationale for EV secretion. Rather than denying regulation or experimental observations, this perspective shifts the burden of proof. Recent discussions have similarly revisited the possibility that vesicle release reflects membrane constraint and homeostatic adjustment rather than directed communication (Muhandiram and Fazeli 2026). The proposed framework does not seek to supplant all existing functional interpretations with a single alternative explanation. Rather, it posits that membrane turnover, proteostatic balance, and energetic constraint establish a fundamental baseline state from which context‐dependent EV functions may arise.

The present perspective examines the quantitative and thermodynamic conditions under which functional claims remain plausible. The mere existence of EVs should not be interpreted as evidence of purpose; instead, functional claims must be substantiated by quantitative, energetic, and organizational constraints that define the cellular default state.

## 2 Thermodynamics Rules Them All

Cell organization is not a passive condition. It is continuously sustained by metabolic flux and energy excess (Ellis 2001). Any maintained asymmetry, gradient, or molecular enrichment requires continuous energy input to persist. Without ongoing metabolic activity, these organized membrane states would relax toward equilibrium. A substantial fraction of these constraints resides in membranes. Lipid asymmetry, curvature stress, electrochemical gradients, and molecular crowding define a physically restricted state that is maintained only because the cell continuously expends energy to keep it away from equilibrium. Membranes are therefore not inert containers. They are dynamic interfaces defined by tension, composition, and potential (Helfrich 1973). If this is adopted, EV biogenesis and maturation can be reinterpreted. Vesicle release is neither a trivial leakage nor solely a genetically encoded export event; rather, it constitutes a regulated transition within the cellular energy landscape. This process provides a mechanism to redistribute, relax, or externalize local membrane constraints. Functional cell‐cell communication may arise as a secondary outcome but should not be presumed to be the default explanation. Membrane curvature, lipid composition, and protein association are interdependent; changes in any of these alter the energetic balance and stability of the local membrane state. Under specific conditions, this coupling can render vesicular geometries energetically preferred, not as a binary decision, but as a continuous modulation of constraint and stability. Protein accumulation at specific membrane regions alters local membrane organization, packing, and curvature stress. These changes can stabilize or destabilize membrane domains, thereby influencing vesicle formation (Peter et al. 2004). The free energy stored in these states represents a real cost in constrained degrees of freedom and an entropic penalty that must be offset for the assembly to persist. Depending on the context, this compensation can stabilize, destabilize, or shift a domain into an alternative assembly.

Consequently, cargo loading and membrane bending should not be conceptualized as sequential steps, but as interdependent processes. Sorting influences curvature, and curvature, in turn, affects sorting; both processes co‐evolve as the system relaxes through accessible states. Thermodynamics does not dictate vesicle formation in a deterministic manner, but instead defines the acceptable frame: what is energetically obtainable, what can persist, and what will collapse without continuous maintenance.

## 3 Vesicle Release Follows the Underlying Chemical Potential Gradient

The mere possibility of an alternative membrane configuration does not imply that it will occur. Transitions between states require the crossing of energy barriers. These transitions are therefore kinetically regulated rather than simply thermodynamically allowed. ESCRT assemblies (Hurley et al. 2025), tetraspanin‐enriched domains (Rubinstein et al. 2025), cytoskeletal remodelling (Loiseau et al. 2016), and lipid‐modifying enzymes (Trajkovic et al. 2008) lower such barriers in a spatially and temporally controlled manner. They bias the system toward specific curvature states and membrane reorganizations. However, these mechanisms do not suspend physical constraints. They operate within the same energetic landscape that defines which configurations are accessible and which remain transient. Vesicle release, therefore, represents a controlled transition within this landscape. It reflects the regulated reconfiguration of a structured membrane state, not the creation of a process outside thermodynamic bounds.

EV release, therefore, cannot be interpreted solely as a signalling output, but may reflect the cell's internal metabolic balance and immediate physical boundary conditions. It derives from metastable membrane states formed by energetic constraints and kinetic selection. The resulting vesicular composition, therefore, does not reflect only active sorting mechanisms, but also preferential stabilization of specific lipid‐protein assemblies under defined physical conditions.

This consideration prompts the question: if EV formation reflects constrained physical states, why is the process so orchestrated? Alterations in lipid composition due to physiological or pathological metabolism, or local protein accumulation, modify the membrane's energetic landscape. The system subsequently relaxes along kinetically accessible pathways that mitigate the imposed imbalance. Analogous to Le Chatelier's principle (Le Chatelier 1884), cellular membranes adapt to alterations in lipid composition or protein accumulation by redistributing local energetic constraints. Unlike classical equilibrium systems, cellular membranes operate under continuous metabolic flux. Vesicle release functions as a relaxation mechanism that temporarily mitigates local membrane and proteostatic stress, though it does not restore equilibrium. Vesicle formation thus constitutes one potential relaxation pathway during sustained metabolic activity. When proteostatic or lysosomal capacity is exceeded, export may serve as a kinetically accessible alternative to intracellular degradation. This framework is particularly pertinent in neurodegenerative disease contexts, where EV release has been proposed as both a clearance mechanism for aggregation‐prone proteins and a possible route for pathological dissemination (Saman et al. 2012; Baixauli et al. 2014). In conditions of aggregate accumulation or impaired autophagic flux, vesicle release may transiently alleviate intracellular constraints. Uptake by neighbouring cells does not necessarily indicate communicative intent but may instead represent a bystander effect of local homeostatic relaxation.

However, EV release is often described as stimulus‐driven and polarized (Raju et al. 2024; Ruiz‐Navarro et al. 2023). This does not contradict the proposed framework. Directed release alone does not establish communication as the primary mechanistic rationale. Energy‐dependent mechanisms act locally to control topology and timing, yet they remain embedded within the same energetic landscape that constrains all membrane states. Any directed secretion must therefore be compatible with the underlying thermodynamic default.

Mechanisms of active cargo sorting, such as ALIX‐associated pathways like ESCRT and Rab GTPase‐dependent trafficking, represent localized energetic investments that enable topological transitions that would otherwise be kinetically suppressed. Their existence does not contradict a thermodynamic baseline; rather, it demonstrates that cells selectively exploit physically permissible membrane configurations. In this sense, ESCRT is not a substitute for physical constraints but rather a regulator that operates within them.

Overall, vesicle biogenesis and release represent regulated transitions within an energetically structured system maintained far from equilibrium. Thermodynamics defines the permissible landscape, while kinetics governs the timing and nature of transitions between states. Perturbations generate flux within this constrained environment, and energy‐dependent mechanisms provide spatial and temporal regulation without altering the basic physical boundaries. Cargo assembly, membrane bending, and vesicle budding are interdependent processes that occur within a unified thermodynamic framework. EV maturation arises at the intersection of energetic bias, kinetic selection, and changes in cellular homeostasis. Vesicle release can thus be interpreted within a thermodynamic framework that reflects membrane turnover and constraints. Functional attribution requires demonstrating deviation from this physically expected background. Vesicle release may be required for intercellular transfer, yet its occurrence alone does not establish that communication constitutes the primary mechanistic or evolutionary rationale of the process.

## 4 Release is Not Function

The mode of vesicle release described above does not necessitate that EVs fulfil the extrinsic functions frequently ascribed to them. The occurrence of an effect is not determined by release alone, but by a sequential chain of events: physical contact and binding, internalization, endosomal escape, delivery of sufficient effector molecules, and ultimately, interaction with a functional intracellular target. In such serial processes, the overall outcome is governed by the limiting step, consistent with Liebig's law of the minimum, formally expressed as E ≤ min (k_1_, k_2_, …, k_n_). Surface‐mediated interactions constitute a distinct mode of EV‐associated function that circumvents several constraints on intracellular delivery. Processes such as ligand‐receptor engagement, membrane docking, fusion events, and protein transfer at contact sites do not require endosomal escape or cytosolic amplification. In these scenarios, parameters such as surface density, binding affinity, and encounter probability become the primary limiting factors, rather than intracellular cargo delivery. These mechanisms are especially relevant in spatially confined environments, such as immune synapses and tumour microenvironments, where local vesicle concentrations and contact durations may exceed the constraints imposed by systemic dilution. Consequently, the quantitative framework proposed here remains applicable, although the limiting steps shift from intracellular transfer efficiency to surface availability and binding kinetics. Biological systems permit amplification. A single delivered mRNA, for instance, can undergo multiple rounds of translation, and viral infection illustrates how rare entry events can expand through replication. Amplification thus reduces the number of initiating events needed to achieve a measurable impact. However, it operates only after successful passage through the preceding steps. It does not remove serial constraints; it amplifies outcomes that have already exceeded the limiting threshold. A single bottleneck can constrain the entire cascade. Enrichment becomes functionally relevant only if it exceeds stochastic incorporation and compensates for dilution across serial transfer steps. High release rates cannot compensate for low uptake efficiency or restricted cytosolic access. Multiple quantitative studies indicate that only a small fraction of EVs that interact with target cells ultimately achieve productive intracellular delivery, with cytosolic access representing a major limiting step rather than a minor technical caveat (Heusermann et al. 2016; Mathieu et al. 2019; Ripoll et al. 2026). Biological systems do not require perfect efficiency. Many cellular processes operate stochastically, and even low‐probability events can be biologically meaningful if amplified by downstream signalling. Evolution selects for sufficient, not maximal, efficiency. However, stochastic operation does not remove quantitative constraints. For EV‐mediated effects to be evolutionarily stable, the combined probabilities of release, encounter, uptake, and cargo engagement must reproducibly exceed background noise. In defined immune synapses or confined microenvironments, such thresholds may plausibly be exceeded. Noise lowers the threshold for relevance but does not abolish the requirement for quantitative plausibility. Disruption of individual steps can generate measurable phenotypes that may be misinterpreted as clear signal transmission. Perturbation phenotypes demonstrate contribution, not evolutionary primacy. Interference with components such as Rab27a may reduce vesicle flux and downstream effects, but this establishes pathway involvement rather than communication as the primary mechanistic rationale for vesicle release (Ostrowski et al. 2010). Such mechanisms are plausible and do not contradict the present system. In addition, the accumulation of free circulating biomolecules on vesicles, also referred to as the EV corona, introduces another modulatory layer that may alter binding or signalling properties. In biological fluids, vesicle surfaces are rapidly modified by the adsorption of circulating proteins, forming an EV corona composed of albumin, complement factors, and immunoglobulins. This extrinsic layer can significantly influence uptake efficiency, receptor interactions, biodistribution, and signalling outcomes. Consequently, functional effects previously attributed to intrinsic EV cargo may, in part, result from corona‐mediated surface remodelling (Tóth et al. 2021; Wolf et al. 2022).

The consistent enrichment of cell‐specific markers on EVs is commonly considered evidence of primary functional specificity. However, recent differential proteomic analyses argue against a general role of CD9, CD81, or CD63 in cargo sorting into EVs (Fan et al. 2023), consistent with a model in which vesicle composition largely reflects membrane topology and local membrane organization rather than directed molecular programming. This observation does not contradict the present model, nor does it exclude selective sorting mechanisms in defined contexts. Rather, it reflects the topological origin and energetic bias of defined membrane domains. Vesicles preferentially incorporate proteins concentrated in regions susceptible to curvature and assembly (Peter et al. 2004; Baietti et al. 2012). Functional specificity may thus emerge as a secondary consequence of membrane organization, rather than serving as the primary driver of vesicle formation. A counterexample illustrates the limits of enrichment‐based specificity. Even classical “exosomal markers” such as CD63 are heterogeneously distributed and not present on all vesicles (Théry et al. 2018). Bulk analyses may therefore report significant enrichment, while single‐vesicle measurements reveal that only a fraction of EVs carry the protein. In such cases, enrichment does not translate into consistent functional specificity but reflects probabilistic incorporation determined by membrane topology. Specificity can thus fail when copy number and distribution remain below functional thresholds. For example, bulk enrichment of CD63‐positive vesicles does not imply that all individual vesicles carry sufficient CD63 molecules to mediate equivalent interactions.

## 5 Stoichiometry Defines the Possibility

**FIGURE 1:**
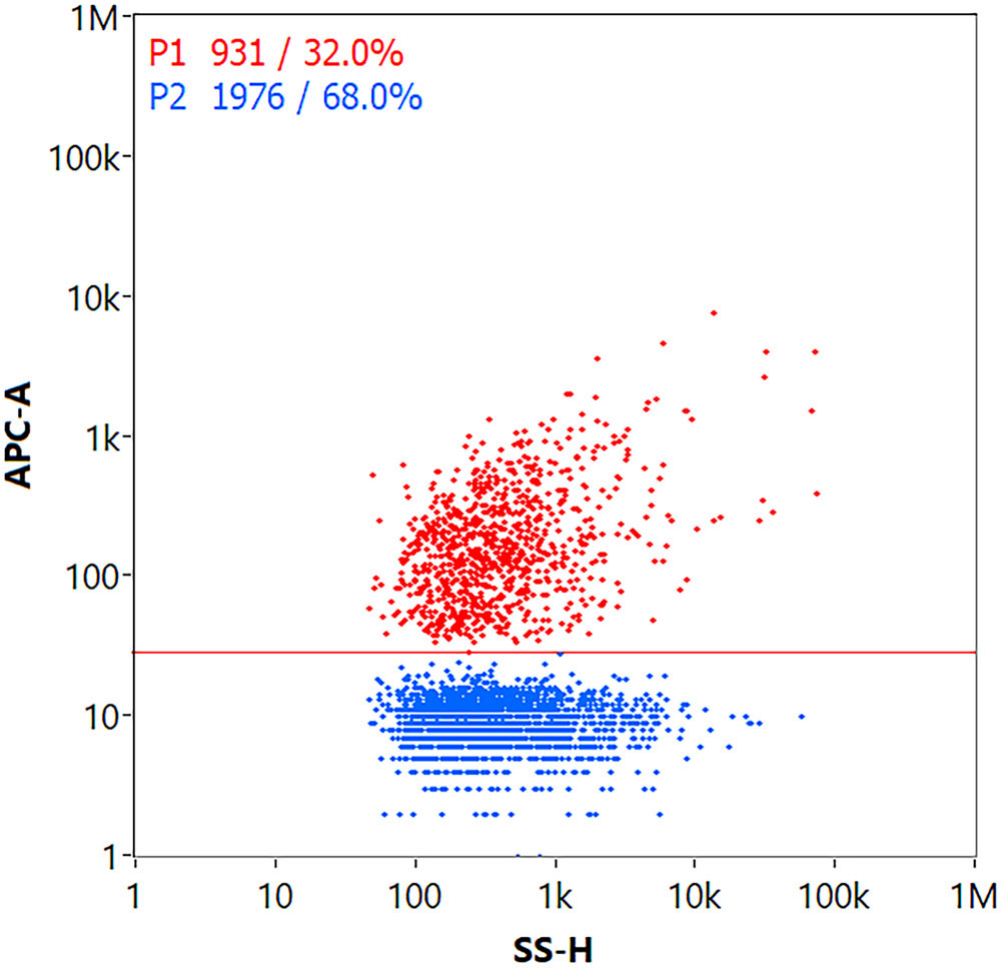
**Single‐vesicle positivity and stochastic partitioning**. Representative single‐vesicle fluorescence plot showing a defined positive fraction (32%). Within a minimal stochastic framework, this corresponds to a low effective mean copy number per vesicle, consistent with a high null fraction at the single‐vesicle level. Enrichment in bulk does not imply uniform distribution of cargo across the population.

Given that a relevant molecule is associated with a vesicle through the mechanisms described above, its distribution and any resulting signaling must obey statistical rules. Molecular cargo is not uniformly distributed, but partitioned into discrete nanoscopic compartments. At low mean copy numbers (λ), stochastic incorporation approximates a Poisson process with P(≥1) = 1−e^−λ^, the limiting case of rare independent events. Differences from this distribution would indicate active enrichment beyond statistical expectation. At low λ, a high zero fraction is therefore expected rather than unusual. In the example shown, 32% of events are CD63‐positive (**Figure** **1**). Conceptually, this means that when only very few molecules are available on average, most vesicles are expected to carry none at all, while only a minority contains one or more copies. Within a minimal stochastic model, this corresponds to λ = −ln(0.68) ≈ 0.38. This value reflects the effective mean copy number per detectable unit and remains below one. This argument applies primarily to rare, potentially functional cargo. For highly abundant tetraspanins, reported in the range of 50–150 copies per vesicle, P(0) approaches zero (Han et al. 2021). Reduced positivity in this case reflects detection limits or population heterogeneity, not true absence. RNA cargo provides a quantitative test case: although often proposed as a major functional mediator, many miRNAs occur at low average copy number per vesicle, imposing stochastic and dilution constraints that must be overcome to achieve reproducible biological impact (Chevillet et al. 2014; Albanese et al. 2021). In recipient cells, endogenous miRNA pools typically exceed these contributions by orders of magnitude. Unless delivery is highly efficient and enriched, cytosolic vesicle‐derived RNA is unlikely to shift the regulatory equilibrium measurably. Claims of gene silencing, therefore, require quantitative reconciliation with cellular stoichiometry.

The topological diversity of vesicle formation does not invalidate this scheme. Direct plasma membrane budding (ectosomal), intraluminal vesicle formation (endosomal), secretory autophagy, or larger membrane‐derived structures differ in their origins and geometries. What they share is that they represent regulated solutions for membrane remodeling. The associated machinery controls surface composition, redistributes or removes molecules, relieves mechanical restrictions, and contributes to organelle quality control.

How, then, can directed cell‐cell communication be reconciled with this view? From an evolutionary perspective, a regulated vesicle flux constitutes a rich pre‐existing resource. Here, communication can emerge when dilution is limited by local proximity, when recipient cells evolve high sensitivity, and when selective pressure stabilizes advantageous interactions. An example is the evolutionary refinement of immune signaling in higher organisms, in which EVs have become instrumental for transmitting specific molecular signals that coordinate immune response. The evolutionary use of a process, however, presupposes its prior existence. It does not account for its origin. In this sense, EVs can be considered consistent with the concept of evolutionary “spandrels” (Gould 1997), in which structural consequences of cellular organization may later acquire functional significance through secondary exaptation.

The central question is not whether EVs can mediate effects, but under which physical and statistical conditions such effects are plausible and reproducible. Functional attribution must be demonstrated relative to this baseline, rather than inferred solely from vesicle detection. The current framework yields experimentally verifiable predictions: if EV‐mediated communication is primary, selective cargo enrichment should consistently surpass stochastic expectations and overcome stoichiometric dilution under physiological conditions. Conversely, if vesicle release primarily reflects constrained membrane remodeling, cargo enrichment will remain limited by copy‐number distributions and heterogeneous topology.

## 6 EVs are Perfect Biomarkers, Aren't They?

If vesicle release reflects membrane turnover and cellular stress, the composition of EVs should correspond to the physiological state of the producing cell. In this context, EVs are conceptually appealing as biomarkers, as they provide real‐time sampling of membrane organization, metabolic status, and protein distribution.

However, this setting also delineates structural limitations. Vesicle populations are inherently heterogeneous, and marker exposure reflects this variation. Preanalytical variables can alter vesicle flux and composition, whereas systemic dilution reduces effective signal strength. Experimental manipulation can perturb membrane tension and metabolic state. Mechanical stress, centrifugation, or prolonged handling may induce additional vesicle release, so measured populations can partly reflect preparation‐induced relaxation rather than the original physiological condition. Low copy numbers introduce statistical noise. Consequently, stoichiometric constraints limit reproducibility at the single‐vesicle level and robustness at the systemic level. Yet the same principles suggest a different perspective. If EVs arise as epiphenomena of membrane turnover, metabolic stress, or altered cellular constraints, they may be imperfect indicators of cellular identity but sensitive indicators of cellular state. Precisely because their composition reflects shifts in membrane dynamics and proteostatic balance, they can report on ongoing perturbations. The modest performance of many EV biomarker candidates is therefore consistent with the underlying boundary conditions: EVs may be limited as stable identity markers yet well‐suited as dynamic state indicators. Because vesicle release reflects membrane turnover, proteostatic load, and metabolic constraint, EV composition may provide a temporally sensitive readout of altered membrane and metabolic states.

## 7 Conclusion

EVs exist and are secreted because membranes are dynamic, and cellular organization requires continuous energy investment. Vesicle release represents a regulated, energetically permissible transition in this setting. In complex biological systems, processes that arise as structural consequences are not evolutionarily inert; they can become substrates for functional refinement once selective pressure stabilizes advantageous interactions. Multiple EV‐associated functions may coexist depending on the biological context. This perspective contends that these functions should be evaluated against an underlying thermodynamic and quantitative baseline rather than inferred solely from vesicle release. Functional communication likely emerges only under specific spatial, quantitative, and evolutionary conditions.

To operationalize this framework, we provide testable criteria (**Table** **1**) that set evidentiary standards for functional attribution across six domains: cargo enrichment, productive delivery, stoichiometric sufficiency, physiological reproducibility, spatial constraints, and surface‐mediated signaling. These criteria distinguish functional EV‐mediated communication from epiphenomenal consequences of vesicle release

**TABLE 1:** Evidence supporting functional attribution.

Criterion	Experimental test	Evidence supporting functional relevance
Cargo enrichment	Compare observed cargo distribution to stochastic models	Reproducible deviation from random incorporation
Surface‐mediated signaling	Measure receptor occupancy, binding kinetics, or contact‐dependent signaling	Surface density and interaction kinetics are sufficient for signaling
Functional delivery	Measure Quantify intracellular delivery and cytosolic access	Detectable delivery under physiological conditions
Stoichiometric sufficiency	Compare delivered cargo to endogenous recipient‐cell pools	Delivered cargo sufficient to measurably alter endogenous molecular balance
Reproducible phenotype	Functional readout without artificial sensitization or supraphysiological dosing	Robust and reproducible effect under physiological conditions
Spatial constraint	Measure encounter probability and local vesicle concentration	Local concentration exceeds systemic dilution constraints

EV‐mediated communication from epiphenomenal consequences of vesicle release.

Characterizing EVs as epiphenomena does not diminish their significance. Rather, it situates their origin within the thermodynamic baseline of cellular homeostasis while linking functional attribution to explicit quantitative and mechanistic criteria.

## Author Contributions

Christian Preußer: conceptualization, writing – original draft, writing – review and editing, visualization. Elke Pogge von Strandmann: conceptualization, writing – review and editing, project administration.

## Funding

This work was supported by the Deutsche Forschungsgemeinschaft (DFG) under Grant (416910386 GRK 2573).

## Conflicts of Interest

None.

## Data Availability

Data sharing not applicable to this article as no datasets were generated or analysed during the current study.
